# Association between EBV serological patterns and lymphocytic profile of SjS patients support a virally triggered autoimmune epithelitis

**DOI:** 10.1038/s41598-021-83550-0

**Published:** 2021-02-18

**Authors:** Filipe Barcelos, Catarina Martins, Ricardo Monteiro, Joana Cardigos, Tiziano Prussiani, Miguel Sítima, Nuno Alves, José Vaz-Patto, Jaime Cunha-Branco, Luís-Miguel Borrego

**Affiliations:** 1 CHRC, Comprehensive Health Research Centre, Lisbon, Portugal; 2 CEDOC, Chronic Diseases Research Center, Immunology, NOVA Medical School|FCM, Universidade Nova de Lisboa, Campo dos Mártires da Pátria, 130, 1169-056 Lisbon, Portugal; 3 Department of Rheumatology, Instituto Português de Reumatologia, Lisbon, Portugal; 4 Department of Rheumatology, Hospital Cuf Descobertas, Lisbon, Portugal; 5 Department of Ophthalmology, Centro Hospitalar de Lisboa Central, Hospital de Santo António Dos Capuchos, Lisbon, Portugal; 6 Department of Ophthalmology, Hospital Cuf Descobertas, Lisbon, Portugal; 7 Department of Rheumatology, Centro Hospitalar de Lisboa Ocidental, Hospital de Egas Moniz, Lisbon, Portugal; 8 Department of Immunoalergy, Hospital da Luz, Lisbon, Portugal

**Keywords:** Autoimmunity, Viral infection, SjÃ¶gren's disease, Immunology, B cells, T cells

## Abstract

Sjögren's syndrome (SjS) is characterized by lymphocytic infiltration of exocrine glands, i.e. autoimmune epithelitis. Lymphocytes are central in SjS pathogenesis, with B-cell hyperactivity mediated by T-cells. B-cells are main targets of Epstein-Barr virus (EBV) infection, a frequently-suggested trigger for SjS. We aimed to evaluate how the EBV infection modulates B and T-cell subsets in SjS, including as controls Rheumatoid arthritis patients (RA) and healthy participants (HC). SjS patients presented decreased CXCR5^+^T-cells, although IL21-secreting Tfh and Tfc cells were increased. Tfc were positively correlated with ESSDAI scores, suggesting their relevant role in SjS pathogenesis. As previously described, SjS patients showed expanded circulating naïve B-cell compartments. SjS patients had a higher incidence of EBV-EA-D-IgG^+^ antibodies, characteristic of recent EBV-infection/reactivation. SjS patients with past infection or recent infection/reactivation showed increased CXCR3^+^Th1 and CXCR3^+^Tfh1 cells compared to those without active infection. SjS patients with a recent infection/reactivation profile presented increased transitional B-cells compared to patients with past infection and increased plasmablasts, compared to those without infection. Our results suggest EBV-infection contributes to B and T-cell differentiation towards the effector phenotypes typical of SjS. Local lymphocyte activation at ectopic germinal centres, mediated by Tfh and Tfc, can be EBV-driven, perpetuating autoimmune epithelitis, which leads to gland destruction in SjS.

## Introduction

Sjögren's syndrome (SjS) is a chronic systemic autoimmune disease, with an estimated prevalence between 0.2–0.5%^1^, affecting predominantly middle-aged women. It is characterized by lymphocytic infiltration of the exocrine glands, referred to as autoimmune epithelitis^2^. Lachrymal and salivary glands (SG) are the most affected glands, originating the hallmark features of xerostomia and xerophthalmia. Extraglandular manifestations are common and can be caused by either lymphocytic infiltration of epithelial tissues, or immune complex disease^3^.

Lymphocytes are central in the pathogenesis of SjS^4^, and a lymphocyte profile with increased naïve B-cells and decrease memory B-cells is typical^5^, reflecting the increased migratory pathway of differentiated B-cells into affected organs^6^. A deviation of B-cell differentiation towards plasma cells has also been described in SjS^7^.

In SjS, T-cells infiltrate affected organs, like the SG, and support hyperactivity of B-cells^8^. In fact, interactions between T-cells and activated B-cells occur in GC-like structures developed in target tissues, such as the SG^9^. Recently, follicular helper T-cells (Tfh) have been addressed as players in SjS pathogenesis. Tfh cells are a major source of interleukin (IL)-21, which mediates B-cell survival and promotes ectopic formation of germinal-centre(GC)-like structures^10^. SjS patients present increased circulating Tfh cells and expanded Tfh differentiation in the SG^11,12^. Tfh cells also express the chemokine receptor X5 (CXCR5), which induces their homing towards lymph nodes, particularly to B-cell sites. Since the expression of CXCR5 has been encountered in CD8^+^ T-cells, the existence of a follicular cytotoxic (CD8^+^) T-cell subset (Tfc) is now accepted, as well as their possible role in the regulation of GC B-cell responses and autoantibody production^13,14^.

The aetiology of SjS is still poorly understood, but the concept of an infectious trigger is widely spread. Virally-triggered autoimmunity in SjS possibly results from an antigen-driven CD4^+^ T-cell activation. Combined with a genetic predisposition to loss of tolerance, this activation process elicits a migration of both CD4^+^ T-cells and B-cells towards exocrine glands, where the expansion and formation of plasma cells occurs^8^. Lymphotrophic viruses, namely Cytomegalovirus (CMV) and Epstein-Barr virus (EBV), are strong candidates for triggering the disease^15^. EBV primary infection occurs in B lymphocytes of the oropharyngeal mucosa, where lytic and latent phases of the viral cycle take place. In the active lytic phase, EBV replicates and propagates, while in the latent phase it remains inactive in B-cells^16^. Viral agents also interfere with T-cell mediated responses^17^. In chronic viral infections, helper T-cells sustain cytotoxic T-cell responses as long as viral antigens persist^18^. Moreover, the proinflammatory Th1 profile, usually present in acute viral infections, is somehow replaced by Tfh in response to viral persistence and prolonged T-cell receptor stimulation^19^.

The implication of EBV in SjS is widely accepted. Mechanisms such as molecular mimicry and genetic susceptibility to EBV infection can overlap with T-cell costimulatory overactivity, impaired EBV-specific T-cell response, cross-reactivity of anti-EBV antibodies or inhibition of B-cell apoptosis, often associated with stimulation-driven polyclonal and monoclonal lymphoproliferation (recently reviewed by Máslínska^20^). Nonetheless, the impact of viral infection on the typical B-cell profile and hypergammaglobulinemia observed in SjS patients needs further clarification. Thus, we aimed to evaluate circulating B and T-cell subsets of SjS patients and to assess their relation to the EBV background of patients.

## Results

### Population

Fifty-seven SjS patients were recruited along with 20 Rheumatoid Arthritis (RA) patients and 24 healthy controls (HC). From our cohort, we assessed EBV serology in 34 SjS patients, 20 RA patients, and 20 HC. Participants' characteristics are presented in Supplementary Table 1.

### T-cell subsets

SjS patients presented lower CD4^+^ T-cell percentages and absolute counts than HC (*p* = 0.002 and *p* < 0.0001, respectively). Accordingly, the absolute counts of CXCR5^+^ Tfh cells were also lower in the SjS group compared to both HC (*p* < 0.0001) and RA patients (*p* = 0.038). However, the percentages of IL21-secreting CD4^+^ T-cells were increased in SjS patients when compared to both RA patients and HC (*p* < 0.0001). Nonetheless, a positive correlation between the percentages of IL21^+^ CD4^+^ T-cells and the percentages of CXCR5^+^ Tfh cells (r = 0.281, *p* = 0.034) was observed.

CD8^+^ T-cells percentages were higher in SjS patients compared to HC (*p* = 0.001), but not absolute counts. Additionally, IL21-secreting CD8^+^ T-cells' were increased in SjS patients when compared to both HC (*p* = 0.029) and RA patients (*p* < 0.001). CXCR5^+^CD8^+^ T-cells were positively correlated with ESSDAI scores (*p* = 0.029, r = 0.430) of SjS patients. Results are summarized in Table 1 and Supplementary Table 2.

**Table 1:** Percentages of T and B-cell subsets in all groups.

Percentages	SjS	RA	HC	*p* value
**T-cell subsets**				
T-cells	74.97 [69.14–78.43]	80.25 [71.59–82.54]	74.71 [70.11–79.57]	0.151
CD4 T-cells	**61.23 [53.09–67.65]**	66.26 [52.80–71.62]	**69.09 [60.83–75.89]**	**0.006**
CXCR5^+^ Tfh	18.44 [14.69–23.56]	20.00 [15.48–25.47]	20.80 [17.38–22.93]	0.502
Tfh1^**#**^	**36.23 [30.18–41.25]**	**28.64 [23.87–38.20]**	31.65 [27.30–35.47]	**0.014**
Tfh17^**#**^	21.15 [16.63–26.72]	24.92 [17.08–29.57]	20.88 [17.57–29.11]	0.405
IL-21^+^	12.41 [8.25–14.92]	8.98 [7.32–11.39]	9.69 [6.32–11.94]	**0.031**
IL-17^+^	2.20 [1.46–3.17]	2.43 [1.20–3.90]	2.40 [1.72–6.74]	0.750
IL-21^+^ IL-17^+^	0.67 [0.50–0.94]	0.71 [0.34–0.94]	0.68 [0.28–1.09]	0.829
CD8 T-cells	**38.40 [31.88–46.92]**	33.74 [28.38–47.21]	**30.90 [23.44–39.17]**	**0.012**
CXCR5^+^ Tfc	2.53 [1.99–3.60]	1.98 [1.42–3.69]	3.44 [1.98–3.80]	0.456
IL-21^+^	4.05 [2.31–5.65]	**2.32 [1.15–3.03]**	2.79 [0.97–4.40]	**0.001**
IL-17^+^	0.89 [0.56–1.40]	1.06 [0.74–1.80]	1.14 [0.75–2.08]	0.217
IL-21^+^ IL-17^+^	0.27 [0.14–0.45]	0.18 [0.09–0.59]	0.33 [0.16–0.75]	0.350
**B-cell subsets**				
B-cells	**9.73 [6.87–13.34]**	**6.38 [4.40–8.60]**	**10.40 [8.57–13.65]**	**< 0.001**
Naïve	66.58 [51.84–77.34]	55.80 [30.28–69.42]	53.01 [43.22–69.21]	**0.030**
Memory	**29.46 [20.23–44.44]**	32.17 [25.38–56.76]	**44.45 [27.37–54.71]**	**0.032**
Unswitched memory	**13.64 [8.91–22.57]**	15.99 [10.80–27.53]	**21.61 [14.57–32.20]**	**0.029**
Switched memory	14.50 [10.00–21.85]	17.41 [13.59–30.69]	19.52 [13.72–26.48]	0.107
Double negative	2.21 [1.51–4.10]	**5.59 [2.33–7.74]**	2.07 [1.44–2.93]	**0.006**
Bm1	**9.60 [5.62–15.63]**	14.27 [11.19–21.66]	13.86 [10.12–22.68]	**0.003**
Bm2	**60.32 [48.75–67.10]**	**48.81 [31.48–63.13]**	53.57 [47.42–61.74]	**0.036**
Bm2′	8.08 [3.72–13.44]	3.97 [1.87–9.91]	5.03 [3.46–8.20]	0.053
Bm3 + 4	1.40 [0.93–3.57]	1.61 [0.82–3.52]	1.19 [0.94–1.97]	0.476
eBm5	8.85 [6.07–12.79]	10.90 [8.28–14.26]	11.76 [8.26–15.69]	0.082
Bm5	**6.87 [4.40–13.07]**	**17.14 [7.42–26.14]**	8.86 [6.81–11.98]	**0.006**

T and B cells subsets' percentages presented in mean ± standard deviation.

Bold numbers highlight the populations that were significantly different. Kruskal–Wallis test was applied for statistical significance.

^**#**^ Tfh1 and Tfh17 are represented as percentages among CXCR5^+^ Tfh cells.

*SjS* Sjögren's syndrome, *RA* rheumatoid arthritis, *HC* healthy controls.

### B-cell subsets

Considering the IgD/CD27 classification, the percentages of IgD^+^CD27^−^ B-cells (naïve) were higher in SjS patients when compared to HC (*p* = 0.028) and RA patients (*p* = 0.043), and SjS patients also presented higher absolute counts of this subset compared to RA (*p* = 0.015). Total memory B-cells (CD27^+^IgD^+/−^) and unswitched memory B-cells (CD27^+^IgD^+^) were lower in SjS patients compared to HC (*p* = 0.001). However, only absolute counts of switched memory B-cells (CD27^+^IgD^−^) were lower in SjS compared to HC (*p* < 0.001), and no differences were observed towards RA patients.

Using the Bm1-5 classification, B-cells were classified as Bm1, Bm2, Bm2′, Bm3 + 4, eBm5, and Bm5 subsets^21^. The percentages of Bm1 cells were significantly lower in SjS compared to RA (*p* = 0.005) and HC (*p* = 0.008), and the absolute counts were also significantly lower in SjS (*p* = 0.002) compared to HC. Bm2 (naïve) and Bm2′ (transitional) percentages were significantly higher in SjS compared to RA patients (*p* = 0.015 for Bm2 and *p* = 0.041 for Bm2′), and Bm2′ absolute counts followed the same trend (SjS vs RA; *p* = 0.003). Lower percentages (*p* = 0.037) and absolute values (*p* < 0.001) of eBm5 cells were found in SjS when compared to HC. As for Bm5 cells, SjS patients presented lower percentages than RA (*p* = 0.011), and lower absolute counts than HC (*p* = 0.001). The results are summarized in Table 1 and Supplementary Table 2.

### EBV serological markers

All patients and controls were negative for anti-VCA IgM and anti-EA IgA, except for 1 HC that presented borderline levels for anti-EA IgA. All samples were positive for anti-VCA IgG, except for 2 SjS patients, who showed negative values for these antibodies. Most patients and HC showed positive values for anti-EBNA IgG (76.5% of SjS; 80.0% of RA and 85.0% of HC), and negative values for anti-VCA IgA (79.4% of SjS; 75.0% of RA and 80.0% of HC), without significant differences between groups. Interestingly, for anti-EA IgG, significant differences were observed between SjS patients and HC (32.4% in SjS; 20.0% in RA; 5.0% in HC). The results are presented in Table 2.

**Table 2:** EBV serological evaluation in SjS, RA and HC.

Group	SjS (n = 34)	RA (n = 20)	HC (n = 20)	*p* value
**IgG antibodies—quantitative assays (positive/negative)**				
EBV CA IgG +	32/2	20/0	20/0	n.s
EBV EA IgG +	11/23	4/16	1/19	0.022*
EBV EBNA IgG +	26/8	16/4	17/3	n.s
**IgA/IgM antibodies—semiquantitative assays (positive/borderline/negative)**				
EBV CA IgA	1/6/27	2/3/15	3/1/16	n.s
EBV CA IgM	0/0/34	0/0/20	0/0 /20	n.s
EBV EA IgA	0/0/34	0/0/20	0/1/19	n.s

Results for the different anti-EBV antibodies in the different patient groups (positive/negative for quantitative assays; positive/borderline/negative for semi-quantitative assays).

*SjS* Sjögren's syndrome, *RA* rheumatoid arthritis, *HC* healthy controls.

*p < 0.05, for SjS versus HC (Fisher’s exact test); n.s., non-significant.

### EBV serological patterns in SjS patients

Recognizing that SjS patients presented an increased prevalence of Anti-EBV EA-D IgG and also an important presence of anti-EBNA IgG, we further divided these patients into 3 subgroups according to the serological EBV profile observed: G1 (n = 18), previous infection (EA-IgG–, EBNA IgG +); G2 (n = 11), recent infection/reactivation (EA IgG + , EBNA IgG + /-), and G3 (n = 5), no serological evidence of active infection (EA IgG–, EBNA IgG–)^20^. Demographic and clinical data are presented in Table 3.

**Table 3:** Characterization of SjS patients and of SjS subgroups with distinct EBV serology.

	Sjögren's syndrome n=34	Distinct EBV serology Sjögren's subgroups		
		G1 EA IgG- EBNA IgG+ n = 18	G2 EA IgG+ EBNA IgG+/- n = 11	G3 EA IgG– EBNA IgG– n = 5
Age (years, median, Min-Max)	57.1 (28.6–74.8)	57.1 (28.6–71.4)	49.1 (29.9–74.8)	63.8 (49.2–67.4)
Age of onset (years, median, Min–Max)	43.7 (24.5–68.3)	43.8 (24.5–58.7)	39.3 (25.3–68.3)	50.9 (36.0–61.2)
Age at diagnosis (years, median, Min-Max)	48.9 (26.7–71.7)	49.3 (26.7–62.0)	48.5 (29.7–71.7)	53.8 (41.6–65.2)
Symptom duration (years, median, Min-Max)	11.9 (1.0–29.5)	13.1 (1.3–29.5)	8.1 (1.0–26.1)	12.8 (5.4–17.8)
Ocular symptoms, n (%)	31 (91.2)	17 (94.4)	10 (90.9)	4 (80.0)
Oral symptoms, n (%)	33 (97.1)	18 (100)	11 (100)	4 (80.0)
Ocular signs, n (%)	22 (64.7)	11 (61.1)	8 (72.7)	3 (60.0)
Oral signs, n (%)	23 (67.6)	15 (83.3)	5 (45.5)	3 (60.0)
Parotid enlargement, n (%)	6 (17.6)	5 (28.8)	1 (9.1)	0 (0.0)
Focus Score ≥ 1, n (%)	23/32 (71.9)	15/17 (88.2)	3/10 (30.0)	5 (100)
Active disease, n (%)	17 (50.0)	9 (50.0)	7 (63.6)	1 (20.0)
ESSDAI (mean, Min–Max)	2.53 (0–14)	2.94 (0–14)	2.27 (0–6)	1.60 (0–4)
ESSDAI ≥ 5, n (%)	5 (14.7)	4 (22.2)	1 (9.1)	0 (0.0)
Extra-glandular disease (ever), *n* (%)	16 (47.1)	8 (44.4)	5 (45.5)	3 (60.0)
Joint symptoms (ever), *n* (%)	13 (38.2)	5 (28.8)	5 (45.5)	3 (60.0)
Skin involvement (ever), *n* (%)	10 (29.4)	4 (22.2)	5 (45.5)	1 (20.0)
Other extraglandular involvment	2 (5.9)	2 (11.1)	0 (0.0)	0 (0.0)
Raynaud's phenomenon	5 (14.7)	3 (16.7)	2 (18.2)	0 (0.0)
SSA (%)	27 (79.4)	13 (72.2)	10 (90.9)	4 (80.0)
SSB (%)	13/30 (43.3)	7/17 (41.2)	3/8 (37.5)	3 (60.0)
ANA ≥ 1/320, n (%)	28 (82.4)	15 (83.3)	8 (72.7)	5 (100)
ANA ≥ 1/640, n (%)	21 (61.8)	12 (66.7)	6 (54.5)	3 (60.0)
Rheumatoid factor, *n* (%)	16/29 (55.2)	9/16 (56.3)	4/9 (44.4)	3/4 (75.0)
Gammaglobulin ≥ 1.6 g/dl, n (%)	11 (32.4)	5 (28.8)	5 (45.5)	1 (20.0)
Therapy (any), n (%)	21 (61.8)	13 (72.2)	5 (45.5)	3 (60.0)
Glucocorticoids, n (%)	12 (35.3)	6 (33.3)	3 (27.3)	3 (60.0)
Hydroxychloroquine, n (%)	12 (35.3)	8 (44.4)	2 (18.2)	2 (40.0)
Imunossupressants, n (%)	6 (17.6)	5 (28.8)	0 (0.0)	1 (20.0)

Patient's characteristics are represented as number of occurrences (n) and percentages (%). Whenever there were missing values, percentages reflect the number of occurrences over the number of patients tested for the item. Ocular evaluation included Schirmer's test and corneal staining score. The oral signs item consisted of a decreased unstimulated salivary flow. Focus score was defined as the number of lymphocyte aggregates (≥ 50 cells) per 4 mm^2^ of glandular area of the biopsy sample. Joint symptoms include arthritis and joint pain of inflammatory origin, but only cases that would score in the articular domain of ESSDAI were considered as extra-glandular disease. Likewise, in some patients skin involvement (which not included xerosis) was not considered as extra-glandular disease if it would not score in the cutaneous domain of ESSDAI. Clinically active disease was defined as activity in any ESSDAI domain, except the hematologic and biologic.

*SjS* primary Sjögren's syndrome, *F* female, *M* male, *y* years, *SSA*/*SSB* Sjögren's syndrome A/B antibody, *ANA* antinuclear antibody, *RF* rheumatoid factor, *ESSDAI*,EULAR Sjögren's syndrome disease activity index.

SjS patients with recent infection/reactivation markers (G2) had earlier disease manifestations and shorter disease duration.

Half of the patients from group G1 and 2/3 of those in group G2 had active disease at the time of recruitment, with G1 patients showing higher ESSDAI scores than G2 patients. The lowest ESSDAI scores were observed in G3 patients. Skin involvement was more frequent in G2 patients. Parotid enlargement and Raynaud's phenomenon were not documented in G3 patients. A higher proportion of G2 patients presented increased gammaglobulin, with higher mean IgG compared to G1 and G3 (1.52 g/dl vs 1.42 g/dl vs 1.29 g/dl, respectively). None of the abovementioned differences reached statistical significance, though.

Regarding T-cells, G1 and G2 patients presented increased CXCR3^+^ CD4^+^ T cells (Th1) cells and CXCR3^+^ CXCR5^+^ CD4^+^ T cells (Tfh1) compared to G3 (Th1: G1vsG3, *p* = 0.121, and G2vsG3, *p* = 0.009; Tfh1: G1vsG3, *p* = 0.003, and G2vsG3, *p* = 0.066).

As for B-cells, transitional Bm2′ cells were augmented in G2 patients compared to G1 (*p* = 0.024). Moreover, plasmablasts (Bm3 + Bm4) were increased in G1 and G2 patients compared to G3 (%, G1vsG3, *p* = 0.088 and G2vsG3, *p* = 0.003; absolute counts, G1vsG3, *p* = 0.020 and G2vsG3, *p* = 0.003).

These data are presented in Table 4 and Supplementary Table 3.

**Table 4:** Immune profile of SjS patients with distinct EBV serology patterns (percentages).

Percentages	G1 EA IgG^−^ EBNA IgG^+^ (n = 18)	G2 EA IgG^+^ EBNA IgG^+/−^ (n = 11)	G3 EA IgG^–^ EBNA IgG^–^ (n = 5)	*p* value
**T-cell subsets**				
T-cells	77.0 [68.7–83.9]	75.6 [71.9–78.4]	74.8 [63.2–76.2]	0.395
CD4 T-cells	59.9 [50.2–63.6]	62.8 [53.0–67.3]	59.7 [47.4–66.7]	0.727
CXCR5^+^ Tfh	17.1 [13.8–24.1]	19.7 [14.2–23.7]	17.4 [13.6–27.7]	0.851
Tfh1^**#**^	37.3 [34.1–41.3]	37.4 [31.3–43.1]	28.2 [19.9–32.7]	0.025*
Tfh17^**#**^	21.6 [16.1–27.8]	18.7 [13.9–26.2]	23.3 [20.2–37.5]	0.168
IL-21^+^	12.4 [8.1–14.5]	13.27 [9.4–15.0]	13.4 [6.9–24.7]	0.857
IL-17^+^	2.42 [1.65–3.38]	2.76 [1.63–3.21]	2.20 [1.52–3.62]	0.882
IL-21^+^ IL-17^+^	0.68 [0.53–1.20]	0.73 [0.64–0.99]	0.58 [0.47–1.12]	0.666
CD8 T-cells	40.1 [36.4–49.8]	37.2 [32.7–47.0]	40.4 [33.4–52.7]	0.716
CXCR5^+^ Tfc	2.40 [2.25–3.38]	2.80 [2.10–3.60]	1.90 [1.50–2.40]	0.152
IL-21^+^	3.94 [2.52–5.30]	4.42 [2.53–8.47]	3.59 [2.04–33.68]	0.698
IL-17^+^	1.04 [0.57–1.38]	1.21 [0.90–1.61]	0.64 [0.42–2.13]	0.467
IL-21^+^ IL-17^+^	0.28 [0.14–0.50]	0.37 [0.23–0.48]	0.34 [0.15–1.14]	0.833
**B cell subsets**				
B cells	9.8 [6.6–11.3]	11.0 [8.3–18.4]	7.7 [5.3–9.5]	0.130
Naïve	66.7 [48.7–74.4]	71.9 [49.4–77.5]	66.6 [50.4–77.1]	0.751
Memory	31.4 [23.3–49.0]	26.5 [19.6–49.7]	29.3 [22.4–47.1]	0.589
Unswitched Memory	15.0 [13.0–24.8]	12.0 [7.9–20.0]	15.0 [6.1–28.5]	0.145
Switched memory	15.3 [10.0–26.8]	14.2 [10.3–22.5]	16.3 [10.9–18.4]	0.978
Double negative	2.11 [1.52–3.06]	2.30 [1.59–3.32]	4.07 [1.04–6.91]	0.803
Bm1	10.0 [7.2–15.7]	5.5 [4.12–10.0]	12.5 [4.5–24.9]	0.203
Bm2	60.5 [45.5–67.1]	60.0 [47.4–64.0]	51.5 [47.7–64.2]	0.831
Bm2’	6.9 [2.3–11.9]	13.6 [6.4–17.4]	5.9 [3.8–18.6]	0.087*
Bm3 + 4	2.21 [1.00–4.24]	2.83 [1.31–4.48]	1.10 [0.74–1.26]	0.043*
eBm5	9.7 [6.0–13.9]	9.2 [7.6–12.8]	11.5 [7.0–12.1]	0.997
Bm5	6.45 [4.67–14.81]	6.24 [4.05–11.45]	8.11 [6.07–14.83]	0.647

Percentage values for all T and B cells subsets in median [minimum–maximum] in SjS patients evaluated for EBV serology.

*SjS* Sjögren's syndrome, *EBV* Epstein-Barr virus.

^**#**^Tfh1 and Tfh17 are represented as percentages among CXCR5^+^ Tfh cells.

*Bold numbers highlight the populations that were significantly different. Kruskal–Wallis test was applied for statistical significance.

## Discussion

Our study aimed to explore the relation between the EBV serological profile of SjS patients and the distribution of circulating B and T-lymphocyte subsets. First, we report interesting differences in follicular T-cell subsets between SjS patients and both HC and RA patients. Despite circulating CXCR5^+^ T cell subsets were decreased in SjS patients, functionally IL21-secreting CD4^+^ (Tfh) and CD8^+^ (Tfc) T cells seem to be more pronounced in these patients. IL21-secreting CD8^+^ T cells (Tfc) were even positively correlated with ESSDAI scores, suggesting their relevant role in SjS pathogenesis. Moreover, we confirmed the enriched circulating naïve B-cell compartment of SjS patients (compared to both control groups, healthy and autoimmune), previously reported in the literature^5^.

The major observation of our study, however, comes from the EBV profile, with SjS patients presenting a greater incidence of EBV-EA-D-IgG positivity, a profile characteristic of recent infection/reactivation of EBV infection. Furthermore, SjS patients with either serological evidence of past EBV infection or recent infection/reactivation presented higher values of CXCR3^+^ CD4^+^ T cells (Th1) and CXCR3^+^ CXCR5^+^ CD4^+^ T cells (Tfh1) compared to those without serological evidence of active infection. Also, the B-cell compartment was distinctive in SjS patients with signs of recent EBV infection/reactivation: showing higher levels of transitional Bm2′ cells compared to patients with past infection and increased plasmablasts, compared to patients without serological evidence of infection.

The factors underlying the onset and development of SjS are still uncertain. Nevertheless, typical immune profiles have been characterized in these patients, which can be relevant to unveil important links to other triggering players in this autoimmune disease. Despite B-cells are the main target for EBV latent infection, T-cells have also a role in this play, and have been studied in autoimmune diseases for which EBV is considered a potential trigger. For instance, EBV-specific CD8^+^ T-cells are increased during B-cell transformation and in the productive viral replication phases of EBV in infected RA^22^ and SLE patients^23^. Additionally, the EBV-specific CD8^+^ T-cell pool is reduced by immunosuppressive therapy^24^.

Our study supports the presence of a promoted follicular T-cell environment in SjS patients, traduced by the increased secretion of IL21 by both CD4^+^ and CD8^+^ T-cells. We found no differences in the percentages of circulating CXCR5^+^ follicular T-cells between groups, and absolute counts for this subset were even decreased in SjS patients, possibly due to the decreased absolute counts of CD4^+^ T-cells observed in these patients. Similar data had been described in the work of Brokstad^25^, which reported no differences for total CXCR5^+^ CD4 T-cell percentages, but only changes in particular subsets of these cells in SjS patients, such as the increase in Tfh-like ICOS^+^PD‐1^+^ cells. Interestingly, in our study, another Tfh-like subset was increased in SjS patients, the IL21^+^ Tfh cells. Indeed, both CD4^+^ and CD8^+^ T-cells were more prone to produce IL21, the Tfh modulating cytokine. If the lower absolute numbers may indicate retention of CXCR5^+^ follicular T-cells at the exocrine glands, as supported by previous studies showing a T-cell predominance in lymphocytic infiltrates of these organs^26^, SjS patients seem to be predisposed to promote Tfh differentiation. Also, when naïve T-cells and salivary gland epithelial cells are co-cultured, Tfh differentiation is observed, i.e. T-cells acquire a classical Tfh phenotype and are able to secrete IL21^27^. Thus, this systemic follicular function may be overexpressed in SjS patients, also as an effect of the local altered interplay. We have previously reported that the ESSDAI score, which is a measure of disease activity in SjS, seemed to be correlated with IL21^+^ CD8^+^ T cells (Tfc) levels^28^. In fact, patients with more active disease present increased circulating Tfc cells, though the causative link between these observations is still to be clarified (i.e. whether higher levels of Tfc cells lead to increased disease severity or, on the contrary, happen in response to disease aggravation).

In addition, the increase in IL21-expressing T-cells resembles the profile of a chronic active viral infection, as proposed by Fahey and collaborators, who showed that viral persistence redirects T-cell differentiation towards the Tfh profile in animal models^19^. Moreover, patients with infectious mononucleosis show an increase in a particular subset of Tfh cells in peripheral blood^29^, which supports our hypothesis that viral triggers may take part in the modulation of the immune responses also in SjS patients^29^.

Interestingly, Fahey and colleagues^19^ proposed that viral-induced Tfh cells deviate from an original Th1 profile. In line with this, we observed that SjS patients with serological evidence for recent infection/reactivation presented increased Th1 and Tfh1 subsets. Thus, the autoimmune background of SjS patients could provide T-cells with alternate activation signals leading them to assume both Th1 and Tfh1 profiles under viral persistence, since it is accepted that the pathogenesis of SjS is mediated by Th1-derived responses^27^.

Strikingly, the implication of Tfc cells in SjS pathogenesis is supported by their increase in patients with higher disease activity. In line with our results, serum levels of IL21 had already been associated with systemic disease activity in SjS^30^, but our results seem to highlight a role for CD8 T-cells in this scenario. Initially, CXCR5^+^ CD8 T-cells were described as early effector memory CD8 T-cells present in B-cell follicles of human tonsils^13^. Recently these cells were implicated in the control of chronic viral infections^29,31,32^. Also, associations between humoral responses and CXCR5^+^ CD8 T-cells^32,33^ were identified, as these cells express co-stimulatory molecules. In fact, increased immunoglobulin production by B-cells occurs when they are co-cultured with CXCR5^+^ CD8 T-cells, suggesting these cells have other immune functions besides cytotoxic activities^33^. Considering that dysregulated humoral responses are present in SjS, Tfc cells, along with Tfh cells, may induce the atypical antibody production of SjS patients. Nevertheless, the major function of the follicular CD8^+^ T-cells may still be limiting the replication of viral agents in B-cell follicles, as these cells show increased cytotoxic capacities^34^.

As for B-cells, it is accepted they are EBV’s main target^35^. Several studies tried to relate SjS pathogenesis with a specific clonality of B-cells. One of the hallmarks of Sjögren’s syndrome is, in fact, the formation of ectopic lymphoid structures (ELS) in the SG. ELS are composed of B-cell/T-cell follicles, supported by networks of stromal follicular dendritic cells, which support ectopic GC reactions^36^. Active EBV infection has been associated with ELS in the SG of SjS patients and appears to contribute to local growth and differentiation of disease-specific autoreactive B-cells^37^. Despite the possibility of an EBV-triggered B-cell proliferation in SjS, EBV-infected memory B-cells were found to express lower levels of self- and poly-reactive antibodies than their uninfected counterparts^38^.

As corroborated by our data, SjS patients present a typical circulating B-cell compartment, enriched in transitional/naïve subsets, in opposition to memory subsets. If we consider the observations from Coleman and colleagues on the effect of EBV in B-cells^39^, we may also suggest a possible role for EBV in the alterations observed in the B-cell compartment of SjS patients. In fact, these authors have recognized that murine transitional B-cells from the spleen can be reservoirs for gammaherpesvirus like EBV, which can remain latent in these cells, prolonging their life span indefinitely. Our results are in line with this hypothesis, as SjS patients with serological evidence of recent infection/reinfection presented higher percentages of transitional Bm2′. Whether this is an effect of EBV or other concomitant viral infection, remains to be elucidated. However, the viral input for this feature of SjS patients can be also supported by our observation that transitional B cells were particularly increased in patients with recent infection/reactivation. We acknowledge that the assessment of the viral genome in different B-cell subsets could clarify this idea.

Regarding the serological EBV markers, we found an increased prevalence of Anti-EBV EA-D IgG in SjS patients, compared to both RA and HC, as described in previous works^37,40^. The anti-EBV EA-D IgG prevalence in our SjS patients (about 33% vs 5% in HC) was very close to the one reported by Pasoto and colleagues^41^ (36% vs 4.5% in HC), which strengths our data, and led us to further assess the immune compartments according to the EBV serological profile of SjS patients. Interestingly, patients with evidence of EBV infection, and particularly those with recent infection/reactivation (EA-D IgG positive) had earlier disease manifestations, but also a distinct immune profile, with a shift towards pro-inflammatory Th1/Tfh1 subsets in the T-cell compartment. Furthermore, SjS patients with evidence of recent infection/reactivation exhibited higher levels of transitional B-cells and plasmablasts, which may traduce the importance of EBV in the modulation of the immune responses in SjS patients, with possible clinical impact, as suggested by an earlier onset of clinical manifestations. The effect of cytotoxic T-cell (CTL) responses, with both CD8^+^ and CD4^+^ T cells, or even other unconventional T cell subsets, may restrict the expansion of latently infected B-cells in long-term carriers or patients with past infection^42,43^. This later immune balance may be the cause for the differences observed in transitional B-cells between SjS patients with recent infection/reinfection and patients with previous infection (G1). Usually increased in SjS, these cells represent a potential EBV reservoir, and are markedly increased in G2 patients, showing however a relapse to lower values in past infections as an effect of an effective immune control happening in these patients (G1).

To better comprehend how EBV profiles modulate immune populations, or whether the changes are SjS-driven, a comparison between SjS patients and HC with similar EBV serological patterns would be very helpful. However, considering the reduced number of HC that could be included in the EBV subgroups G1 (3 HC) and G2 (1 HC), such analysis was not possible. In the future, we aim to better address this question, extending the study to a larger cohort, with more SjS patients, but also healthy controls.

Clinical differences between groups were also difficult to assess due to the small size of our patients' groups. The ESSDAI score in both groups of patients with positive EBV serology (G1 and G2) was higher than in EBV-negative patients (G3). G1 patients had a non-significantly higher ESSDAI compared to G2 patients, in line with a recent report by Sanosian^44^, who didn't find distinct ESSDAI scores in anti-EBV EA-positive compared to anti-EBV EA-negative patients. No differences were found in specific clinical ESSDAI domains between G1 and G2 groups, contrasting to the report of Pasoto^41^, who found higher articular activity in anti-EBV EA-positive patients. Nevertheless, the greater frequency of active disease in our G2 patients may suggest an influence of recent EBV infection/reactivation in disease activity status, which may be further supported by the higher levels of IgG observed in this group, despite no statistical significance was achieved. Furthermore, our observations on the B-cell compartment are in line with this, as the increased transitional Bm2′ and plasmablasts observed in the EBV infection/reactivation group also suggests a higher B-production and differentiation on these patients, possibly contributing in parallel for the increased gammaglobulin levels and greater disease activity.

Interestingly, in our study, IgA and IgM EBV-antibodies behaved similarly in SjS, RA and HC, with a predominance of negative samples for these biomarkers in all groups. As for VCA IgM, an acute-phase marker, despite it may be present in different viral scenarios, no differences were expected in SjS according to previous reports^45^.

In other systemic autoimmune diseases, particularly SLE, increased levels of IgA antibodies against the two lytic antigens studied (VCA and EA) have been reported^45,46^. Literature is scarce for this evaluation in SjS patients, nonetheless, both VCA-IgA and EA-IgA seem to be less present in SjS that in other autoimmune conditions such as SLE. Along with a few previous studies^45,46^, our data suggest a lower mucosal immune response against EBV in SjS, compared to other autoimmune conditions, with anti-EBV IgA antibodies prevalence similar to HC. Whether this corresponds to a better or worst control of latent infections in SjS remains to be elucidated.

To our knowledge, our study is the first to report an association between EBV serological patterns and the immune profile of SjS patients, despite EBV EA (early antigen) had already been correlated with autoantibodies production^40^. Although we observed no further differences in the clinical manifestations of SjS patients according to their EBV serology, we were able to identify distinct immune profiles according to the EBV serological pattern. Still, we must recognize the low number of patients considered and the absence of other confirmatory methodologies for the effective viral infection. Nonetheless, our data support the idea that different EBV serological profiles affect circulating B and T-cells in SjS patients. In fact, a more active serological background, as the ones observed in groups G1 and G2, may suggest a viral influence in the immune system driving it to the more pro-inflammatory scenario observed in SjS patients when compared to both other autoimmune conditions or healthy controls.

Other authors have supported the hypothesis that reactivation in the lytic phase of EBV infection promotes immunological dysfunction in SjS^37^. Considering our results, we also believe special attention should be given to the group of SjS patients with serological evidence for recent infection/reactivation, which present a pro-inflammatory profile, with increased Th1/Tfh1 ratio cells along with elevated transitional B-cells and increased plasmablast differentiation.

We acknowledge limitations in our study, such as the absence of standard molecular biology assays to confirm EBV infection. In future studies, it would be relevant to assess not only serology, but also EBV viral load, and eventually other viruses with potential impact in SjS development, such as CMV. Also, our study was performed exclusively in peripheral blood, and we realize it may not properly reflect the numbers and interactions of immune cells at exocrine glands. For instance, SG biopsies would not only clarify the hypotheses on cell traffic between affected organs and the circulating lymphocyte pool but would also allow us to prove the presence of EBV in such organs.

Nevertheless, from our results, it is possible to suggest that EBV plays a role in inducing B and T-cells towards an effector phenotype. EBV enters the replicating phase in the exocrine glands, where this facilitated interaction between EBV antigens and effector T-cells might lead to a breakdown of tolerance. The ensuing autoimmune response mediated by effector B and T-cells might lead to a localized lymphocyte activation with the formation of ectopic GC or GC-like structures. This process, mediated by Tfh and Tfc, can thus perpetuate the autoimmune epithelitis and result in gland destruction.

Our work provides a new perspective on how EBV might be involved in lymphocytic alterations known to be a feature in SjS. Clarifying the role of follicular CD4 and CD8 T-cells in the context of viral infection can be of great value in confirming a viral-triggered autoimmune response in SjS, but a specific strategy for the characterization of these cells in peripheral blood and target organs is still needed.

Our study can also constitute a starting point for approaching the role of CXCR5^+^ and IL21^+^ CD8 T-cells (Tfc) in the context of autoimmunity. The association between CXCR5^+^ CD8 T-cells and disease activity in SjS observed in our study may be an indicator of their involvement in the pathophysiology of autoimmune epithelitis. In the current scenario where Tfc cells involvement in autoimmune pathologies is yet to be elucidated, our study pioneers the association of Tfc cells with human autoimmunity and paves the way for further studies regarding Tfc cells in autoimmune diseases. Indeed, considering the possible pathogenic role of EBV in the pathogenesis of SjS, therapies directed towards the interaction between EBV and activated effector T-cells and B-cells could halt the EBV-triggered lymphocytic activation, and have a relevant clinical applicability.

## Methods

### Population

In this study, we included SjS patients classified according to the 2016 American College of Rheumatology(ACR)/European League Against Rheumatism (EULAR) criteria^47^, and Rheumatoid Arthritis (RA) patients classified according to the 2010 ACR/EULAR criteria^48^. Patients were consecutively recruited, considering as additional exclusion criteria for SjS the use of B-cell-depleting therapies, and for RA patients the presence of xerostomia or xerophthalmia, as well as the use of any biologic disease-modifying anti-rheumatic drug. Disease activity in SjS was evaluated with the EULAR SjS disease activity index (ESSDAI)^49^. Clinically active disease was defined as activity in any ESSDAI domain, except the hematologic and biologic.

The healthy control group (HC) consisted of women without symptoms or signs of xerostomia or xerophthalmia, or any history of autoimmune rheumatic diseases, selected from the Ophthalmology outpatient clinic of Hospital CUF Descobertas.

Informed consent was obtained from all participants. The study was approved by the Ethics committees of both recruiting institutions, and NOVA Medical School Ethics Committee (no. 17/2016/CEFCM).

### Flow cytometry procedures

For the immunophenotyping protocols, peripheral blood samples collected in EDTA-coated tubes were processed and analyzed within 24 h of collection. A pre-validated panel of monoclonal antibodies (mAbs) was used for the characterization of T and B-cell subsets, including CD3, CD4, CD8, CD19, CD24, CD27, CD38, CCR6, CCR7, CXCR3, CXCR5, Anti-IgD, and Anti-IgM. A lyse-wash protocol was performed for both T and B-cell characterization. A lyse-no wash single platform strategy was used to obtain absolute counts of all cell subsets(BD Trucount tubes BD Biosciences, San Diego CA, USA). All samples were acquired in a 4-color cytometer (BD FACS-Calibur, BD Biosciences).

CellQuest Pro (BD Biosciences) software was used for acquisition and analysis purposes and Infinicyt 2.0 (Cytognos S.L., Salamanca, Spain) software was also used for more differentiated subset analysis.

**Figure 1:**
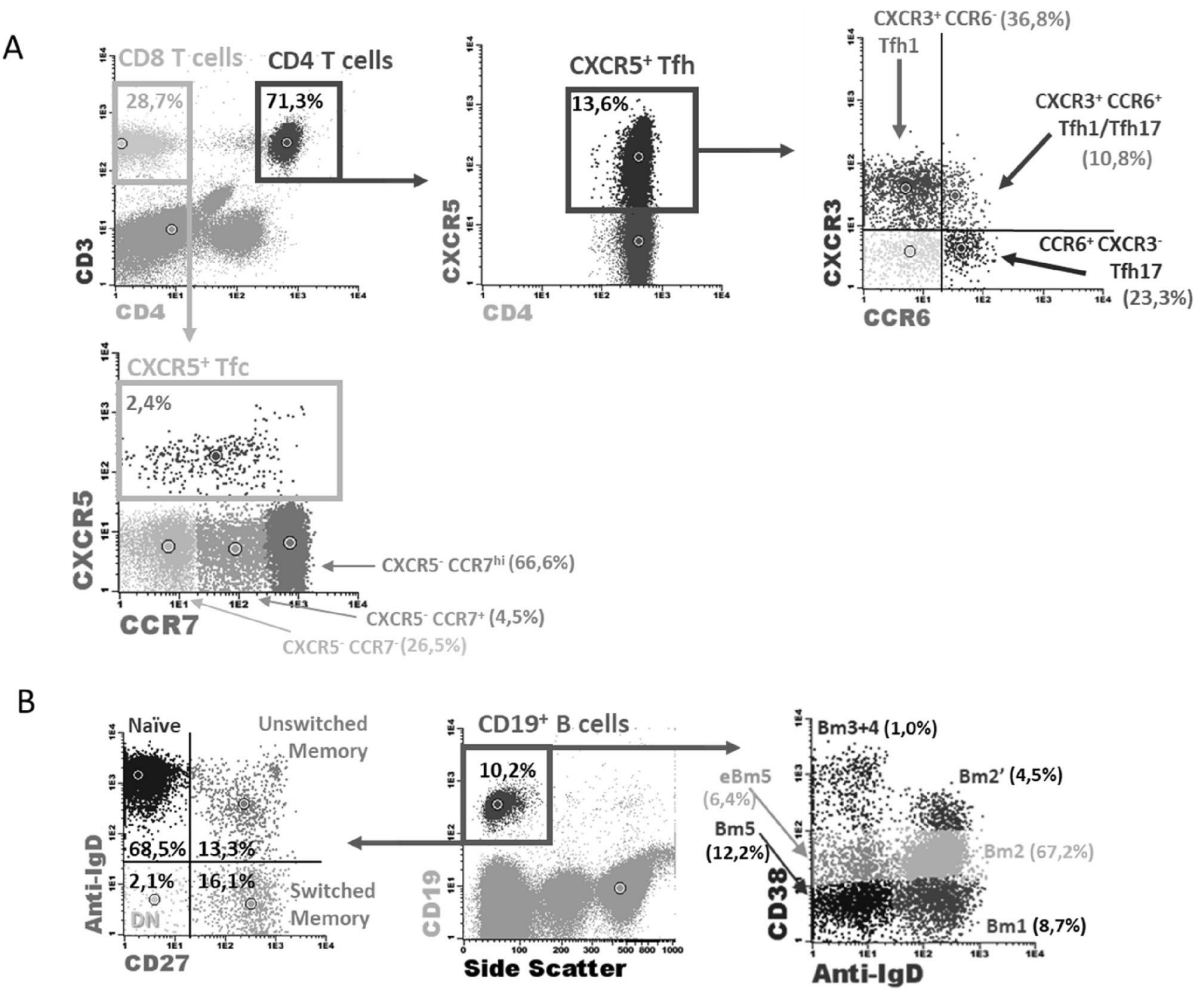
Gating strategy for the identification of circulating T and B-cell subsets. (**A**) Gating strategy for the identification of CD4 T-cells (CD3^+^ CD4^+^ lymphocytes): CXCR5^+^ CD4 T-cells were identified (Tfh), and within this subset, according to the expression of CXCR3 and CCR6, Tfh1 (CXCR3^+^ CCR6^−^), Tfh17 (CXCR3^−^ CCR6^+^) and Tfh1/Tfh17 (CXCR3^+^ CCR6^+^) cells were identified. CD8 T-cells were identified as the CD3^+^ CD4^−^ population of lymphocytes. Within CD8 T-cells, CXCR5^+^ cells were identified (Tfc), but also CXCR5^−^ cells were characterized according to the expression of CCR7 (negative, positive and high positive). (**B**) Gating strategy for the identification of B-cells (CD19^+^ lymphocytes). Using the expression of IgD and CD27 cells were divided in naïve (IgD^+^ CD27^−^), unswitched memory (IgD^+^ CD27^+^), switched memory (IgD^−^ CD27^+^) and double negative (IgD^−^ CD27^−^). Using the Bm1-5 classification, considering IgD and CD38, B-cells were divided in Bm1 (CD38^−^ IgD^+^), Bm2 (CD38^+^ IgD^+^), Bm2′ (CD38^hi^ IgD^+^), Bm3 + 4 (CD38^hi^ IgD^−^), eBm5 (CD38^+^ IgD^−^) and Bm5 (CD38^−^ IgD^−^). +: positive;  - : negative; hi: high; Tfh—Follicular helper T cells; Tfc—Follicular cytotoxic T cells; DN—Double negative.

Whenever appropriate, fluorescence-minus-one control tubes were prepared to assess the positivity of dimer expressions. The subsets analyzed, and the respective gating strategies, are displayed in Fig. 1. Within T-cells, we characterized CD4^+^ and CD8^+^ (CD4-) subsets, including CXCR5^+^ Tfh and Tfc cells, and the Tfh1 and Tfh17 profiles, according to the expression of CXCR3 and CCR6, respectively. B-cells' subsets were addressed according to the classical IgD/CD27 classification, and the Bm1-5 classification, often used in autoimmunity settings^21^.

### Functional assays for the evaluation of IL21 production by T-cells

Heparinized peripheral blood samples were used to assess IL21 and IL-17 production by CD4^+^ and CD8^+^ T-cells.

**Figure 2:**
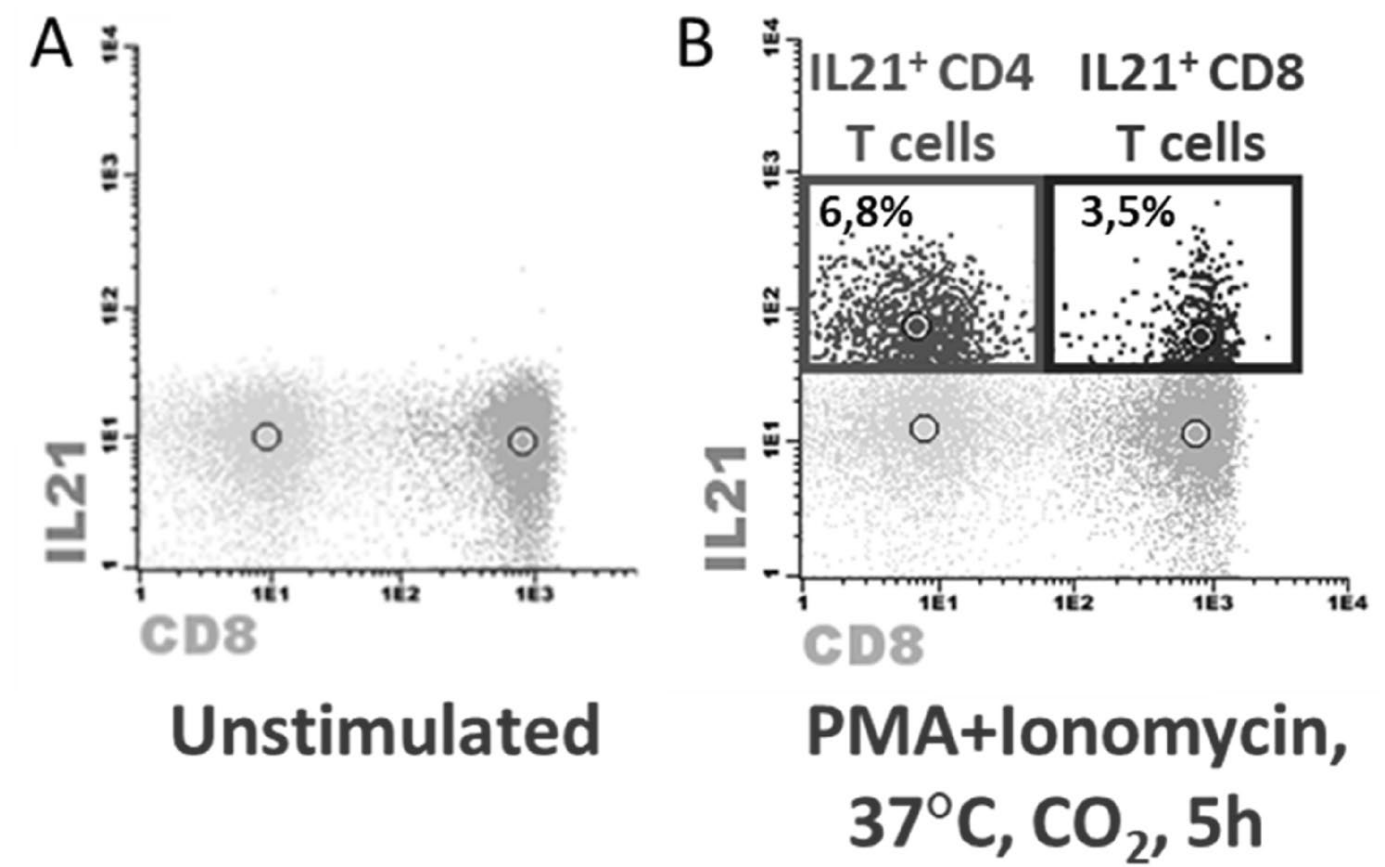
Gating Strategy for the identification of circulating CD4 and CD8 T-cells secreting IL-21 after stimulation. After identifying T-cells according to the expression of CD3 in the lymphocyte gate, CD4 T-cells were identified as the CD3^+^ CD8^−^ subset and CD8 T-cells as the CD3^+^ CD8^+^. (**A**) Expression of IL-21 and IL-17 in CD4 and CD8 T-cells after a 5-h incubation period with no stimulation. (**B**, **C**). Expression of IL-21 and IL(**-**)7 in CD4 and CD8 T-cells after a 5-h stimulation with PMA and ionomycin, in the presence of brefeldin A. Unstimulated controls were used for each sample, to assess the levels of positivity for IL-21 and IL-17.

In brief, cells were stimulated with PMA and ionomycin, for 5 h at 37ºC in a 5% CO_2_ atmosphere in the presence of brefeldin-A. After stimulation, cells were lysed, washed and incubated with anti-CD3 and anti-CD8 mAbs for surface staining. For intracellular stain, cells were treated according to the protocol defined by the manufacturer for the BD Fixation/Permeabilization Solution Kit with BD GolgiPlug™ (*BD Biosciences*) and then marked with anti-IL21 and anti-IL-17 mAbs, after cell fixation and permeabilization. For each patient, stimulated and unstimulated tubes were run in parallel to assure proper stimulation and staining controls. Gating strategy is presented in Fig. 2, with IL21^+^ (IL17^−^) cells being identified within CD8^+^ and CD8^−^ T cells, respectively considered as Tfc and Tfh cells.

### EBV serological markers

Enzyme-linked immunosorbent assays (ELISA) were used for the assessment of IgG, IgA and IgM antibodies (Abs) against EBV antigens (Ags). All ELISA kits were obtained from Euroimmun (*Euroimmun, Luebeck, Germany*) and used according to the manufacturers’ instructions. The following Abs for EBV Ags were determined: IgG for diffuse early Ag (EA-D), IgG for viral capsid Ag (VCA), IgG for nuclear Ag-1 (EBNA1), IgA for EA-D, IgA for VCA and IgM for VCA. All tests for IgG Abs were quantitative, while IgA and IgM were semiquantitative. In quantitative assays, sample concentration was determined using 3-point calibration curves constructed with ELISA-Logit software, available at https://ednieuw.home.xs4all.nl/Calibration/Logit/Logit.htm (V24May2017). The cut-off level for all IgG antibodies assayed was 20 RU/ml. For semiquantitative assessments, a single calibrator was determined in triplicate per assay. The ratio sample/calibrator was used to assess positivity levels (Negative: ratio < 0.8; Borderline: ratio ≥ 0.8 to < 1.1; Positive: ratio ≥ 1.1). Patients were randomly assigned to undergo EBV serology evaluation.

### Statistics

Graph Pad Prism™ 6.0 (*Graph Pad Software, San Diego, CA, USA*) was used for statistical analysis. The normality of data sets was assessed using D'Agostino & Pearson omnibus and Shapiro–Wilk normality tests. ANOVA and Kruskal–Wallis tests were made for multiple analyses among groups, followed by Dunn's multiple comparisons test. When a significant difference was found, comparisons were done using Unpaired Student’s t-test with Welch’s correction or Mann–Whitney test, for every two groups. For categorical variables, Fischer’s or Chi-square tests were applied to assess differences between groups. Statistical significance was considered for *p* values < 0.05.

### Ethics approval

This study was approved by the Ethics committee of *Hospital Cuf Descobertas,* 8/09/2014, Ethics committee of *Instituto Português de Reumatologia,* 3/07/2015 and NOVA Medical School Ethics (No. 17/2016/CEFCM).

### Informed consent

All patients have signed informed consent to participate according to the Declaration of Helsinki.

## Supplementary Information

Supplementary Table 1.

Supplementary Table 2.

Supplementary Table 3.
