# Supplementary material for: Association between EBV serological patterns and lymphocytic profile of SjS patients support a virally triggered autoimmune epithelitis
Source: Sci Rep. 2021 Feb 18;11:4082. doi: 10.1038/s41598-021-83550-0 (PMC7893064; doi:10.1038/s41598-021-83550-0)
Supplement: Supplementary file 1 — Supplementary Table 1. [file 41598_2021_83550_MOESM1_ESM.docx]

**Supplementary Table 1** SjS, RA patients and HC characteristics.

|  | SjS (*N* = 57) | RA (*N* = 20) | HC (*N* = 24) |
| --- | --- | --- | --- |
| Female gender, n (%) | 56 (98.2) | 16 (80.0) | 24 (100) |
| Age, years  median (Min-Máx) | 60.6  (28.6 – 78.3) | 56.7  (30.30-78.0) | 51.3  (38.9 – 63.2) |
| Age of onset, years | 47.1  (24.5-68.3) | 38.6  (22.2-61.9) | - |
| Age at diagnosis, years | 53.8  (26.7-77.2) | 42.5  (22.9-64.0) | - |
| Duration of disease, years | 11.3  (1.0-29.5) | 10.2  (1.5-38.2) | - |
| ESSDAI, median (Min-Máx) | 2  (0-14) |  |  |
| Ocular symptoms, *n* (%) | 54 (94.7) |  |  |
| Oral symptoms, *n* (%) | 55 (96.5) |  |  |
| Ocular signs, *n* (%) | 36 (63.2) |  |  |
| Oral signs, *n* (%) | 42 (73.7) |  |  |
| Focus Score ≥1^a^ | 43/55 (78.2) |  |  |
| Parotid enlargement (ever), *n* (%) | 8 (14.0) |  |  |
| Extraglandular disease (ever)^b^,  *n* (%) | 23 (40.4) |  |  |
| Joint symptoms (ever)^b^, *n* (%) | 24 (42.1) |  |  |
| Skin involvement (ever)^c^, *n* (%) | 18 (31.6) |  |  |
| Other extraglandular involvement (ever), *n* (%) | 5 (8.8) |  |  |
| Raynaud's phenomenon (ever), *n* (%) | 8 (14.0) |  |  |
| SSA, *n* (%) | 38 (66.7) |  |  |
| SSB, *n* (%) | 18/50 (36.0) |  |  |
| ANA ≥1/320 | 45 (78.9) |  |  |
| Rheumatoid Factor, *n* (%) | 24/50 (48.0) |  |  |
| Gammaglobulin ≥1.6 g/dL, *n* (%) | 14 (24.6) |  |  |
| Therapy (any), *n* (%) | 32 (56.1) |  |  |
| Glucocorticoids, *n* (%) | 19 (33.3) |  |  |
| Hydroxychloroquine, *n* (%) | 20 (35.1) |  |  |
| Immunosuppressants, *n* (%) ^d^ | 10 (17.5) |  |  |

**Table legend:**

The patient’s characteristics are represented as number of occurrences (n) and percentages (%).

^a^ Defined as the presence of 1 or more dense aggregates of 50 or more lymphocytes, per 4 mm^2^ of glandular area.

^b^ Joint symptoms include arthritis and joint pain of inflammatory origin, but only cases that would score in the articular domain of ESSDAI were considered as extraglandular disease.

^c^ Skin involvement considered was polymorphous exanthema, purpura or vasculitis, and subacute cutaneous lupus lesions,.

^d^ Any of the following: Methotrexate, Azathioprine, Leflunomide, or Cyclosporine.

* SjS vs RA, Fisher’s exact test

^#^ Brown-Forsythe ANOVA test, with Dunnett's T3 multiple comparisons test showing significant difference between SjS and HC

SjS, Sjögren’s syndrome; RA, Rheumatoid arthritis; HC – Healthy controls; ESSDAI, European Sjögren’s syndrome disease activity index; SSA/SSB, Sjögren’s syndrome A/B antibody; ANA, antinuclear antibody.
