# Supplementary material for: Association between EBV serological patterns and lymphocytic profile of SjS patients support a virally triggered autoimmune epithelitis
Source: Sci Rep. 2021 Feb 18;11:4082. doi: 10.1038/s41598-021-83550-0 (PMC7893064; doi:10.1038/s41598-021-83550-0)
Supplement: Supplementary file 2 — Supplementary Table 2. [file 41598_2021_83550_MOESM2_ESM.docx]

**Supplementary table 2 - Absolute counts of T and B-cell subsets in all groups.**

| **Absolute Counts** | **SjS** | **RA** | **HC** | **p-value** |
| --- | --- | --- | --- | --- |
| T-cell subsets | | | | |
| T-cells | **1180**  **[778 - 1778]** | 1369  [1229 - 1742] | **1777**  **[1426 - 2155]** | **0.001** |
| CD4 T-cells | **746**  **[431 – 1073]** | 908  [691 – 1122] | **1108**  **[1010 – 1408]** | **< 0.001** |
| CXCR5^+^ Tfh | **134**  **[82 – 203]** | 181  [137 – 219] | **241**  **[221 – 284]** | **< 0.001** |
| Tfh1 | 51  [25 - 72] | 51  [39 - 73] | **80**  **[48 - 102]** | **0.002** |
| Tfh17 | **28**  **[16 - 46]** | 41  [27 - 58] | **53**  **[37 - 71]** | **<0.001** |
| IL-21^+^ | 84  [52 – 122] | 91  [63 – 105] | 113  [71 – 167] | 0.157 |
| IL-17^+^ | **13**  **[8 -26]** | 22  [12 – 34] | **30**  **[21 – 46]** | **0.001** |
| IL-21^+^ IL-17^+^ | 5  [3 – 8] | 6  [3 – 8] | 7  [5 – 13] | 0.143 |
| CD8 T-cells | 434  [321 – 643] | 528  [336 – 667] | 533  [394 – 753] | 0.225 |
| CXCR5^+^ Tfc | 3  [2 – 5] | 3  [1 – 7] | 5  [3 – 6] | 0.222 |
| IL-21^+^ | **18**  **[10 – 27]** | **9**  **[5 – 15]** | 13  [6 – 26] | **0.041** |
| IL-17^+^ | **3**  **[2 – 7]** | 5  [3 – 9] | **6**  **[4 – 12]** | **0.037** |
| IL-21^+^ IL-17^+^ | 1  [1 – 2] | 2  [1 – 2] | 2  [1 – 4] | 0.164 |
| **B-cell subsets** | | | | |
| B-cells | **177**  **[97 - 261]** | **133**  **[83 – 153]** | **252**  **[173 - 393]** | **<0.001** |
| Naïve | 108  [61 – 191] | **68**  **[31 – 97]** | 108  [61 - 191] | **0.002** |
| Memory | 47  [29 - 75] | 46  [24 - 57] | **106**  **[65 – 141]** | **<0.001** |
| Unswitched Memory | 22  [12 - 38] | 18  [13 - 30] | **57**  **[32 - 81]** | **<0.001** |
| Switched Memory | 22  [15 - 37] | 20  [13 - 36] | **45**  **[37 - 76]** | **<0.001** |
| Double negative | 3  [2 - 9] | 7  [2 - 11] | 4  [2 - 9] | 0.318 |
|  | | | | |
| Bm1 | 18  [10 – 27] | 19  [11 - 25] | **38**  **[19 - 62]** | **0.001** |
| Bm2 | 101  [57 - 164] | **59**  **[22 - 84]** | 130  [97 - 214] | **<0.001** |
| Bm2’ | 11  [6 – 29] | **4**  **[2 – 10]** | 12  [7 - 30] | **0.005** |
| Bm3+4 | 3  [1 – 5] | 2  [1 – 3] | 4  [2 - 5] | 0.089 |
| eBm5 | 15  [9 – 23] | 11  [7 – 17] | **27**  **[19 - 45]** | **<0.001** |
| Bm5 | **11**  **[8 – 19]** | 14  [9 - 28] | **24**  **[14 - 35]** | **0.002** |

**Table legend:**

T and B cells subsets absolute counts, presented in median [25^th^ – 75^th^ percentile].

***** Bold numbers highlight the populations that were significantly different. Kruskal-Wallis test was applied for statistical significance.

**^#^** Tfh1 and Tfh17 are represented as percentages among CXCR5^+^ Tfh cells.

SjS, Sjögren's Syndrome. RA, Rheumatoid Arthritis. HC, Healthy Controls.
