# Supplementary material for: Association between EBV serological patterns and lymphocytic profile of SjS patients support a virally triggered autoimmune epithelitis
Source: Sci Rep. 2021 Feb 18;11:4082. doi: 10.1038/s41598-021-83550-0 (PMC7893064; doi:10.1038/s41598-021-83550-0)
Supplement: Supplementary file 3 — Supplementary Table 3. [file 41598_2021_83550_MOESM3_ESM.docx]

**Supplementary Table 3** – Immune profile of SjS patients with distinct EBV serology patterns (absolute counts)

| **Absolute Counts** | **G1**  **EA IgG ^-^ EBNA IgG^+^**  **(n=18)** | **G2**  **EA IgG^+^ EBNA IgG^+/ -^**  **(n=11)** | **G3**  **EA IgG^–^ EBNA IgG^–^**  **(n=5)** | **p -value** |
| --- | --- | --- | --- | --- |
| T-cell subsets | | | | |
| T-cells | 1126 [780 - 2145] | 1263 [755 - 1772] | 930 [814 - 1638] | 0.925 |
| CD4 T-cells | 755 [395 - 1227] | 823 [395 - 1159] | 607 [423 - 903] | 0.892 |
| CXCR5^+^ Tfh | 134 [88 - 189] | 140 [82 - 207] | 119 [68 - 209] | 0.774 |
| Tfh1 | 49 [35 - 67] | 60 [35 - 72] | 34 [17 - 52] | 0.283 |
| Tfh17 | 27 [15 - 43] | 28 [14 - 44] | 28 [19 - 64] | 0.949 |
| IL-21^+^ | 90 [45 - 132] | 93 [51 - 122] | 64 [45 - 159] | 0.943 |
| IL-17^+^ | 14 [10 - 32] | 20 [10 - 29] | 9 [8 - 35] | 0.764 |
| IL-21^+^ IL-17^+^ | 7 [2 - 13] | 7 [4 - 8] | 3 [3 - 9] | 0.662 |
| CD8 T-cells | 501 [319 - 1072] | 597 [333 - 636] | 346 [305 - 811] | 0.783 |
| CXCR5^+^ Tfc | 15 [9 - 27] | 15 [5 - 22] | 8 [5 - 17] | 0.237 |
| IL-21^+^ | 20 [12 - 47] | 18 [10 - 37] | 23 [7 - 121] | 0.967 |
| IL-17^+^ | 5 [2 - 11] | 4 [3 - 8] | 3 [2 - 10] | 0.681 |
| IL-21^+^ IL-17^+^ | 2 [1 - 3] | 1 [1 - 3] | 2 [1 - 4] | 0.809 |
| B-cell subsets | | | | |
| B-cells | 169 [103 - 259] | 187 [168 - 261] | 112 [62 - 209] | 0.334 |
| Naïve | 97 [55 - 188] | 126 [73 - 186] | 61 [42 - 145] | 0.355 |
| Memory | 50 [36 - 112] | 64 [33 - 92] | 40 [19 - 52] | 0.306 |
| Unswitched Memory | 26 [19 - 61] | 24 [12 - 38] | 15 [8 - 26] | 0.156 |
| Switched Memory | 23 [15 - 37] | 32 [21 - 52] | 19 [8 - 33] | 0.250 |
| Double negative | 3 [2 - 8] | 3 [2 - 9] | 3 [2 - 7] | 0.769 |
| Bm | | | | |
| Bm1 | 19 [10 - 29] | 16 [9 - 19] | 13 [8 - 22] | 0.370 |
| Bm2 | 91 [49 - 178] | 101 [74 - 139] | 57 [36 - 115] | 0.326 |
| Bm2’ | 11 [5 - 22] | 24 [11 - 29] | 8 [4 - 33] | 0.132 |
| **Bm3+4** | **3 [2 - 5]** | **7 [2 - 8]** | **1 [1 - 2]** | **0.009*** |
| eBm5 | 15 [9 - 26] | 18 [13 - 24] | 13 [4 - 26] | 0.434 |
| Bm5 | 10 [8 - 20] | 18 [8 - 27] | 9 [6 - 23] | 0.608 |

**Table legend:**

Absolute values for all T and B cells subsets presented in median [minimum – maximum] in SjS patients evaluated for EBV serology.

***** Bold numbers highlight the populations that were significantly different. Kruskal-Wallis test was applied for statistical significance.

SjS, Sjögren's syndrome. EBV, Epstein-Barr virus
